# Increase in *bla*_NDM_ among Carbapenemase-Producing, Carbapenem-Resistant Enterobacterales, United States, 2016–2023

**DOI:** 10.3201/eid3206.251404

**Published:** 2026-06

**Authors:** Uzma Afroz Ansari, Davina Campbell, Joshua M. Brandenburg, Nadezhda Duffy, Julian E. Grass, Alice Y. Guh, Joseph D. Lutgring, Christopher A. Elkins, Maria Karlsson, Amy S. Gargis, HAIC MuGSI Working Group

**Affiliations:** Centers for Disease Control and Prevention, Atlanta, Georgia, USA (U.A. Ansari, D. Campbell, J.M. Brandenburg, N. Duffy, J.E. Grass, A.Y. Guh, J.D. Lutgring, C.A. Elkins, M. Karlsson, A.S. Gargis); Chenega Government Missions Solutions, Chesapeake, Virginia, USA (M. Karlsson)

**Keywords:** antimicrobial resistance, bacteria, antibiotics, carbapenem-resistant Enterobacteriaceae, microbial sensitivity tests, public health surveillance, United States

## Abstract

We report an increase of *bla*_NDM_ among carbapenemase-producing, carbapenem-resistant Enterobacterales collected in the United States through the Emerging Infections Program’s Multi-site Gram-negative Surveillance Initiative. Among 1,288 isolates identified, the percentage harboring *bla*_NDM_ increased from 5.4% in 2016 to 39.8% in 2023.

Carbapenem-resistant Enterobacterales (CRE) are an urgent public health threat. In 2022, an estimated 13,387 CRE infections occurred among hospitalized patients in the United States (*1*). Different mechanisms may contribute to carbapenem resistance, but carbapenemase-producing CRE (CP-CRE) are of particular concern. Carbapenemase genes confer broad resistance to β-lactam antimicrobial drugs and are often located on mobile genetic elements, enabling them to spread between bacterial species. Metallo-β-lactamases (MBLs), including New Delhi metallo-β-lactamase (NDM)–producing CRE, are especially problematic because few antimicrobial drugs have activity against them (*2*). We report an increase in *bla*_NDM_ among CP-CRE collected through the Centers for Disease Control and Prevention’s Emerging Infections Program (EIP) Multi-site Gram-negative Surveillance Initiative.

The EIP Multi-site Gram-negative Surveillance Initiative conducts active, population- and laboratory-based surveillance of CRE (*3*–*5*). During 2016–2023, a total of 10 EIP sites participated in surveillance for carbapenem-resistant *Enterobacter cloacae* complex, *Escherichia coli*, and *Klebsiella* species (*K. pneumoniae*, *K. oxytoca*, and *K. aerogenes*) in isolates from a usually sterile site or from urine. Each site submitted a convenience sample of isolates to the Centers for Disease Control and Prevention for testing, including in-house reference broth microdilution incorporating an MBL screen, species identification, and real-time PCR for carbapenemase genes (*3*). We tested all isolates for *bla*_KPC_*, bla*_NDM_*,* and *bla*_OXA-48-like_ genes; we also tested isolates with a positive MBL screen for *bla*_VIM_ and *bla*_IMP_ (*4*). We defined CP-CRE as isolates positive for a carbapenemase gene by real-time PCR and resistant to ertapenem, imipenem, or meropenem as determined by broth microdilution and Clinical and Laboratory Standards Institute breakpoints (Appendix Table) (*6*). Using the Pearson χ^2^ test, we compared the percentages of *bla*_NDM_ among CP-CRE collected in 2016 and 2023. Annual reports with epidemiologic, clinical, and laboratory data are available online (*5*).

Among 1,288 CP-CRE identified, *bla*_KPC_ was the most common carbapenemase gene detected among all confirmed CP-CRE during 2016–2023 (72.7%, 937 isolates), followed by *bla*_NDM_ (20.6%, 265 isolates), *bla*_OXA-48-like_ (7.5%, 96 isolates), *bla*_IMP_ (0.6%, 8 isolates), and *bla*_VIM_ (0.5%, 7 isolates) (Figure); 25 (1.9%) isolates harbored >1 carbapenemase gene (Appendix Table). The percentage of *bla*_NDM_ isolates increased from 5.4% in 2016 (n = 6) to 39.8% in 2023 (n = 99) (p<0.00001). Conversely, *bla*_KPC_ decreased from 92.8% in 2016 (n = 103) to 53.0% in 2023 (n = 132) (p<0.00001) (Figure). Again comparing 2016 and 2023, we observed an increase in *bla*_NDM_ among *E. coli* (1.8% to 22.8%), *Klebsiella* spp. (3.6% to 11.6%), and *E. cloacae* complex (0.6% to 5.2%) (Table). NDM-producing Enterobacterales were more resistant to β-lactam combination agents than were *K. pneumoniae* carbapenemase–producing Enterobacterales (Appendix Table).

**Figure:**
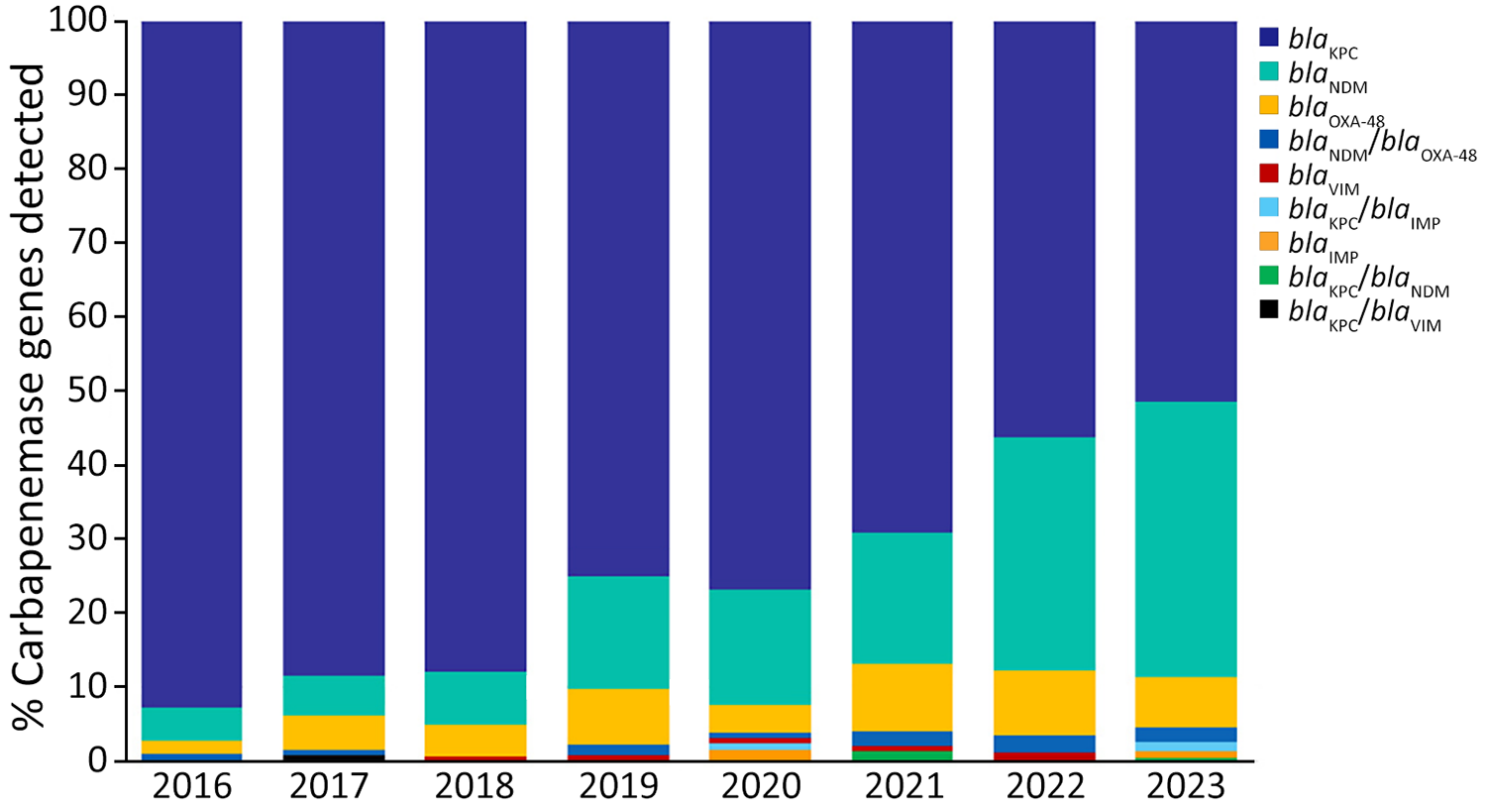
Percentage of carbapenemase genes detected by real-time PCR among 1,288 CP-CRE chosen for a study analyzing an increase in *bla*_NDM_ among carbapenemase-producing, carbapenem-resistant Enterobacterales, United States, 2016–2023. IMP, imipenemase; KPC, *Klebsiella pneumoniae* carbapenemase; OXA-48, oxacillinase; NDM, New Delhi metallo-β-lactamase; VIM, Verona integron-encoded metallo-β-lactamase.

**Table:** Frequency of *bla*_NDM_ by species from study investigating increase in *bla*_NDM_ among CP-CRE, United States, 2016–2023*

Year	Total no. CP-CRE isolates	No. (%) isolates			
		All* bla*_NDM_†	*Escherichia coli*	*Klebsiella *spp.	*Enterobacter cloacae *complex
2016	111	6 (5.4)	2 (1.8)	4 (3.6)	0
2017	149	9 (6.0)	5 (3.4)	4 (2.7)	0
2018	166	12 (7.2)	6 (3.6)	5 (3.0)	1 (0.6)
2019	145	24 (16.6)	13 (9.0)	9 (6.2)	2 (1.4)
2020	134	22 (16.4)	13 (9.7)	6 (4.5)	3 (2.2)
2021	153	32 (20.9)	17 (11.1)	10 (6.5)	5 (3.3)
2022	181	61 (33.7)	27 (14.9)	23 (12.7)	11 (6.1)
2023	249	99 (39.8)	57 (22.9)	29 (11.6)	13 (5.2)
Total	1288	265 (20.6)	140 (10.9)	90 (7.0)	35 (2.7)

*Data are updated for accuracy and may differ from online reports (accessed 2025 Aug 19) (*5*). CP-CRE, carbapenemase-producing, carbapenem-resistant Enterobacterales. †Includes isolates carrying* bla*_NDM_ (n = 245), dual mechanism* bla*_NDM_+*bla*_OXA-48_ (n = 17) ,and* bla*_NDM_+*bla*_KPC_ (n = 3).

We report a notable shift in the type of carbapenemase genes among a convenience sample of 1,288 CP-CRE collected in the United States during 2016–2023. Although *bla*_KPC_ remained the most common carbapenemase gene, we observed a decrease in the proportion of *bla*_KPC_ coupled with an increase in *bla*_NDM._ This shift was most striking among *E. coli*, with *bla*_NDM_ representing 73% of all carbapenemase-producing *E. coli* in 2023; in contrast, we observed *bla*_NDM_ among only 14.3% of carbapenemase-producing *E. coli* in 2016.

The increase of *bla*_NDM_ is alarming given that NDM-producing CRE are more resistant than other CRE isolates (*7*). Furthermore, newer β-lactam combination agents, including ceftazidime-avibactam, meropenem-vaborbactam, and imipenem-relebactam, are ineffective against MBL enzymes, and treatment options are limited (*2*,*8*). Aztreonam/avibactam and cefiderocol have demonstrated in vitro efficacy, but resistance has also been reported (*7*).

Our study is limited because it is based on a convenience sample of isolates from population-based surveillance and might be affected by sampling bias. We collected isolates from 10 EIP sites and national trends may not be extrapolated based on these data. This study is not able to determine whether there are changes in the incidence of NDM-producing CRE, however, our findings align with recent reports of rising NDM-positive CRE in New York City and among the Antimicrobial Resistance Laboratory Network (*9*,*10*). Whole-genome sequencing analysis is needed to determine gene variants and whether increases are driven by specific sequence types.

In conclusion, we report a concerning increase in *bla*_NDM_ among a convenience sample of CP-CRE collected across 10 EIP sites in the United States. Further investigation is needed to assess if this is a nationwide trend, to analyze epidemiologic data comparing characteristics of patients infected with *bla*_NDM_-CRE and those with *bla*_KPC_, and to examine whole-genome sequencing data to determine if the observed increase is related to clonal expansion. Our findings should alert clinicians to the increase in *bla*_NDM_ and encourage mechanism testing in clinical laboratories.

Appendix Additional information for study analyzing an increase in *bla*_NDM_ among carbapenemase-producing, carbapenem-resistant Enterobacterales, USA, 2016–2023.
