# Supplementary material for: Increase in blaNDM among Carbapenemase-Producing, Carbapenem-Resistant Enterobacterales, United States, 2016–2023
Source: Emerg Infect Dis. 2026 Jun;32(6):1028–30. doi: 10.3201/eid3206.251404 (PMC13245226; doi:10.3201/eid3206.251404)
Supplement: Appendix — Additional information for study analyzing an increase in blaNDM among carbapenemase-producing, carbapenem-resistant Enterobacterales, USA, 2016–2023. [file 25-1404-Techapp-s1.pdf]

Article DOI: http://doi.org/10.3201/eid3206.251404

EID cannot ensure accessibility for supplementary materials supplied by authors. Readers who have difficulty accessing supplementary content should contact the authors for assistance.

Increase in *bla*$_{\text{NDM}}$ among Carbapenemase-Producing, Carbapenem-Resistant Enterobacterales, United States, 2016–2023

Appendix

Appendix Table. In vitro activity of antibiotics tested against CP-CRE in the United States, EIP MuGSI: 2016–2023*

Species (n) and Antibiotics	CLSI Interpretive Criteria		
	%S	%I	%R
All Enterobacterales (1,288)			
Amikacin	76.4	11.1	12.5
Ampicillin	0.2	0.1	99.7
Ampicillin-sulbactam (n = 834)†	0.2	0.1	99.6
Aztreonam	6.7	1.1	92.2
Cefepime‡	6.1	0.0	83.5
Cefotaxime	1.5	1.5	97.0
Cefoxitin	10.9	10.3	78.7
Ceftazidime	3.8	2.7	93.5
Ceftazidime-avibactam	75.7	0.0	24.3
Ceftolozane-tazobactam (n = 1211)†	3.6	3.7	92.7
Ceftriaxone	1.3	0.4	98.3
Ciprofloxacin	13.0	5.7	81.2
Colistin	0.0	92.2	7.8
Ertapenem	0.1	0.1	99.8
Gentamicin	62.3	7.1	30.6
Imipenem	4.7	11.5	83.9
Imipenem-relebactam (n = 755)†	64.1	3.3	32.6
Levofloxacin	17.6	7.6	74.8
Meropenem	12.7	10.2	77.0
Meropenem-vaborbactam (n = 955)†	73.3	2.8	23.9
Minocycline (n = 646)†	62.4	17.6	20.0
Piperacillin-tazobactam	0.9	2.5	96.7
Tetracycline	56.8	9.8	33.4
Tigecycline	96.1	3.1	0.8
Trimethoprim-sulfamethoxazole	22.4	0.0	77.6
Tobramycin	36.2	8.1	55.7
All NDM-Enterobacterales (265)§			
Amikacin	65.3	5.7	29.1
Ampicillin	0.4	0.0	99.6
Ampicillin-sulbactam (n = 234)†	0.4	0.0	99.6
Aztreonam	22.6	2.6	74.7
Cefepime‡	0.8	0.0	98.9
Cefotaxime	0.4	0.0	99.6
Cefoxitin	1.9	0.0	98.1
Ceftazidime	0.4	0.0	99.6
Ceftazidime-avibactam	1.1	0.0	98.9
Ceftolozane-tazobactam (n = 259)†	1.2	0.0	98.8
Ceftriaxone	0.4	0.0	99.6
Ciprofloxacin	14.0	4.2	81.9
Colistin	0.0	95.8	4.2
Ertapenem	0.0	0.0	100.0

Species (n) and Antibiotics	CLSI Interpretive Criteria		
	%S	%I	%R
Gentamicin	52.5	1.9	45.7
Imipenem	1.1	3.4	95.5
Imipenem-relebactam (n = 222)†	3.2	2.3	94.6
Levofloxacin	20.0	4.5	75.5
Meropenem	2.6	0.8	96.6
Meropenem-vaborbactam (n = 244)†	9.0	9.0	82.0
Minocycline (n = 203)†	60.6	19.2	20.2
Piperacillin-tazobactam	0.4	0.0	99.6
Tetracycline	38.5	4.9	56.6
Tigecycline	96.6	2.6	0.8
Trimethoprim-sulfamethoxazole	17.0	0.0	83.0
Tobramycin	26.4	7.9	65.7
All KPC-Enterobacterales (n = 937)¶			
Amikacin	78.9	13.2	7.9
Ampicillin	0.2	0.1	99.7
Ampicillin-sulbactam (n = 534)	0.2	0.2	99.6
Aztreonam	0.7	0.3	98.9
Cefepime‡	5.9	0.0	80.7
Cefotaxime	0.7	1.8	97.4
Cefoxitin	13.1	12.9	74.0
Ceftazidime	3.0	3.4	93.6
Ceftazidime-avibactam	95.3	0.0	4.7
Ceftolozane-tazobactam (n = 868)†	2.3	4.7	93.0
Ceftriaxone	0.1	0.3	99.6
Ciprofloxacin	12.3	6.1	81.6
Colistin	0.0	90.6	9.4
Ertapenem	0.1	0.1	99.8
Gentamicin	64.4	9.3	26.4
Imipenem	3.6	12.3	84.1
Imipenem-relebactam (n = 475)†	94.9	2.3	2.7
Levofloxacin	16.4	8.4	75.1
Meropenem	11.5	13.0	75.5
Meropenem-vaborbactam (n = 640)	98.1	0.2	1.7
Minocycline (n = 391)†	64.7	15.9	19.4
Piperacillin-tazobactam	0.7	3.4	95.8
Tetracycline	63.5	11.2	25.3
Tigecycline	95.8	3.3	0.9
Trimethoprim-sulfamethoxazole	23.5	0.0	76.5
Tobramycin	37.2	8.2	54.5

*Interpretations were determined according to Clinical and Laboratory Standards Institute document M100-S35. 2025. (Clinical and Laboratory Standards Institute, Wayne, PA; https://clsi.org/). I, intermediate R, resistant S, susceptible

†Over time, select antibiotics were added or removed from broth microdilution panels and were only tested on a subset of isolates.

‡Cefepime does not have an intermediate category, but has a susceptible-dose-dependent category (CLSI doc M100-S35)

§Dual-mechanism bla_{NDM} / $bla_{OXA\text{-}48\text{-}like}$ and bla_{KPC} / bla_{NDM} included (n = 17 and n = 3, respectively)

¶Dual-mechanism bla_{KPC} / bla_{IMP}, bla_{KPC} / bla_{NDM} and bla_{KPC} / bla_{VIM} included (n = 4, n = 3 and n = 1, respectively)
